# Efficacy of maintenance electroconvulsive therapy in recurrent depression: a case series

**DOI:** 10.1192/j.eurpsy.2023.1761

**Published:** 2023-07-19

**Authors:** G. Guerra Valera, Ó. Martín Santiago, M. Esperesate Pajares, Q. D. L. de la Viuda, A. A. Gonzaga Ramírez, C. Vallecillo Adame, C. de Andrés Lobo, T. Jiménez Aparicio, N. Navarro Barriga, B. Rodríguez Rodríguez, M. Fernández Lozano, M. J. Mateos Sexmero, A. Aparicio Parras, M. Calvo Valcárcel, M. A. Andreo Vidal, P. Martínez Gimeno, M. P. Pando Fernández, M. D. L. Á. Guillén Soto

**Affiliations:** 1 Psychiatry; 2 Psychology, HCUV, Valladolid, Spain

## Abstract

**Introduction:**

Maintenance electroconvulsive therapy (mECT) is an option in the treatment of affective disorders which progress is not satisfactory. It is certainly neglected and underused during the clinical practice.

**Objectives:**

To evaluate the efficacy of mECT in reducing recurrence and relapse in recurrent depression within a sample of three patients.

**Methods:**

We followed up these patients among two years since they received the first set of electroconvulsive sessions. We applied the Beck Depression Inventory (BDI) in the succesives consultations for evaluating the progress.

**Results:**

The three patients were diagnosed with Recurrent Depressive Disorder (RDD). One of them is a 60 year old man that received initially a cycle of 12 sessions; since then he received 10 maintenance sessions. Other one is a 70 year old woman that received initially a cycle of 10 sessions; since then she received 6 maintenance sessions. The last one is a 55 year old woman that received initially a cycle of 14 sessions; since then she received 20 maintenance sessions.

All of them showed a significant reduction in depressive symptoms evaluated through BDI and clinical examination. In the first case, we found a reduction in the BDI from the first consultation to the last that goes from 60 to 12 points; in the second case, from 58 to 8 points; and in the last case, from 55 to 10 points. The main sections that improved were emotional, physical and delusional.

As side-effects of the treatment, we found anterograde amnesia, lack of concentration and loss of focus at all of them.

**Conclusions:**

We find mECT as a very useful treatment for resistant cases of affective disorders like RDD.

It should be considered as a real therapeutic option when the first option drugs have been proved without success.

**Disclosure of Interest:**

None Declared

